# Continuous Decline of *Toxoplasma gondii* Seroprevalence in Hospital: A 1997–2014 Longitudinal Study in Paris, France

**DOI:** 10.3389/fmicb.2018.02369

**Published:** 2018-10-05

**Authors:** Nicolas Guigue, Lucie Léon, Samia Hamane, Maud Gits-Muselli, Yann Le Strat, Alexandre Alanio, Stéphane Bretagne

**Affiliations:** ^1^Laboratoire de Parasitologie-Mycologie, AP-HP, Groupe Hospitalier Saint-Louis, Lariboisière, Fernand-Widal, Paris, France; ^2^Santé Publique France, French National Public Health Agency, Saint-Maurice, France; ^3^Université Paris Diderot, Sorbonne Paris Cité, Paris, France

**Keywords:** *Toxoplasma gondii*, serology, prevalence, immunocompromised patient, France

## Abstract

**Background:** The protozoan *Toxoplasma gondii* presents a risk for reactivation of latent cysts in immunocompromised patients. Anti-*T. gondii* antibodies are therefore usually screened before chemotherapy or transplantation to propose prophylactic measures against this parasite. We analyzed the results obtained in our hospital to study the epidemiological trend of *T. gondii* infection.

**Methods:** We collected all the anti-*Toxoplasma* antibody titers from January 1, 1997 to December 31, 2013 using the Platelia IgG ELISA assay (Bio-Rad). The results were classified as positive when titers reached a concentration of ≥10 UI/ml. Only the first result obtained at entry for each patient was considered. *T. gondii* seroprevalence was estimated using a multivariate logistic regression model accounting for age, sex, and year in which the sample was collected.

**Results:** A total of 21,480 patient samples were analyzed. The seroprevalence continuously decreased over time, from 64.5% in 1997 to 54.7% in 2013 (i.e., an average of 1.3% per year, *p* < 0.001). The decrease was 5.0% per year for patients <20 years. After 2013, the model predicts that the seroprevalence would continuously decrease. We also observed a higher proportion of seropositive men than women in the 15- to 45-year-old group (58.5% versus 52.0%, *p* < 10^-3^).

**Conclusion:** The overall seroprevalence of toxoplasmosis at our hospital showed an accelerating downward trend over 17 years. The reason for this continuous decline is likely associated with the lower parasite presence within meat. Thus, although young immunocompromised patients are increasingly less at risk of reactivation in the near future, older immunocompromised patients will remain at high risk of reactivation. The reasons of the higher prevalence in men remain to be explored.

## Introduction

*Toxoplasma gondii* is a protozoan parasite whose cycle involves cats as the definite host, and any warm-blooded animals including humans as intermediate hosts. Human infection occurs mainly by ingesting tissue cysts from undercooked meat or by ingesting food or drink contaminated by environmental oocysts (Cook et al., 2000; Dubey and Jones, 2008). After a first contamination, which is usually asymptomatic, cysts remain alive in various tissues, especially those of the brain and muscles (Butler et al., 2013). This explains the life-sustaining presence of anti-*Toxoplasma* immunoglobulin G (IgG) antibodies. Thus, infection is usually diagnosed based on the presence of residual IgG antibodies to *T. gondii* which are detectable 2 weeks after infection. With few exceptions (Bossi and Bricaire, 2004), severe, symptomatic toxoplasmosis occurs in only two human populations: the fetus and the immunocompromised patient.

For the fetus, the risk is related to the first infection during pregnancy (Wallon et al., 2013). Given this risk, the French health authorities launched a prevention program in 1978, which is still on-going, based on repeated mandatory monthly serology tests for every pregnant woman in order to detect any possible seroconversion^1^. As a result, cross-sectional seroprevalence studies based on the screening of pregnant women are routinely published (Berger et al., 2009; Nogareda et al., 2014).

For the immunocompromised patient, the strategy differs since the risk is not the primo-infection but rather the reactivation of latent cysts secondary to immunosuppressive treatments (Martino et al., 2000). Due to this risk of *Toxoplasma*-related life-threatening events, screening for the presence of anti-*Toxoplasma* antibodies before any immunosuppressive therapy is recommended when the expected resultant immunosuppression is high (Gea-Banacloche et al., 2009; Ullmann et al., 2016). In hematology, a positive test means the patient is at risk of latent cyst reactivation (Martino et al., 2000). A screening strategy and a work-up using PCR for these identified populations can then be recommended in, for example, cases of febrile neutropenia (Bretagne et al., 2000; Martino et al., 2005). In kidney transplant recipients, a pre-transplantation check-up includes a *Toxoplasma* serology test in order to examine the risk of reactivation in countries with a high risk of toxoplasmosis^2^, whereas this recommendation is restricted to heart transplantation recipients in countries with a low risk of toxoplasmosis (Fischer et al., 2013). Similarly, HIV-infected patients are screened in order to identify their susceptibility to toxoplasmosis reactivation (Kaplan et al., 2009). The practice of wide-range screening in our hospital leads to a continuous collection of anti-*Toxoplasma* antibody results in both sexes and in a wide variety of ages. This screening has afforded us the ability to investigate toxoplasmosis epidemiology over 17 consecutive years using the same commercial kit on an otherwise rarely studied population compared to pregnant women or women of childbearing age (Pappas et al., 2009).

## Patients and Methods

### Study Design

We collected anti-*T. gondii* IgG antibody results from January 1, 1997 to December 31, 2013 through our laboratory registry. When several tests were available for a given patient, only the first result was considered. The demographic data available were age and sex. Saint-Louis Hospital is a 650-bed tertiary university hospital with major clinical activities in onco-hematology, renal transplantation, and infectious diseases, but without maternity.

This non-interventional study did not change the usual procedures and did not need any additional sampling. The analysis was based on existing data from previously performed tests according to physicians’ prescriptions. According to the French Health Public Law (CSP Art L1121-1.1), such protocols do not require approval by an ethics committee and is exempt from the otherwise mandatory informed consent requirements.

### Serological Tests

The same anti-*Toxoplama* ELISA test (Platelia toxo IgG, Bio-Rad) was used throughout the study. Based on thresholds recommended by the manufacturer, the results were classified as positive when titers reached a concentration of ≥10 UI/ml, as equivocal when between 6 and 9 UI/ml, and as negative when <6 UI/ml. Equivocal samples were tested by the Toxo-Screen DA IgG agglutination assay (BioMérieux) as a second routine test for IgG. Sera with values >40 (expressed as dilution titer) were scored as positive, as equivocal when at 40, and as negative if <40. Due the cessation of commercialization of the Toxo-Screen DA IgG assay in May 2012, a second commercial test (Toxo-HAI, Fumouze) based on hemagglutination was used in its stead as of this date. Tested samples were defined as positive if values reached >1/80, as equivocal when at 1/80, and as negative if <1/80. When the Platelia toxo IgG was equivocal and one of the other tests also equivocal or positive, the result was considered as positive.

### Statistical Analysis

A logistic regression model was employed to estimate *T. gondii* seroprevalence as a function of age, sex, and date of sample collection, including age as a continuous covariate. We used fractional polynomials to estimate the relationship between *T. gondii* infection and age (Royston et al., 1999). All analyses were performed using Stata 14.2 and R 3.3.2.

## Results

**FIGURE 1:**
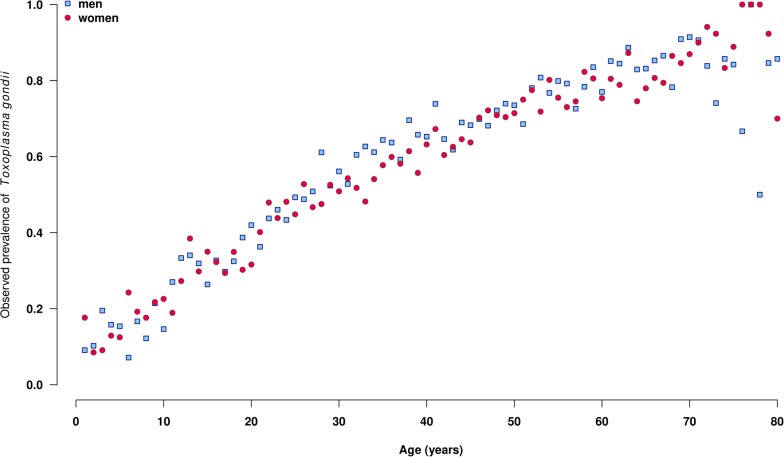
Observed prevalence of the presence of anti-*Toxoplasma gondii* antibodies by age, among men (red points, *n* = 11,994) and women (white points, *n* = 9,486), Saint-Louis Hospital, France, 1997–2013.

**FIGURE 2:**
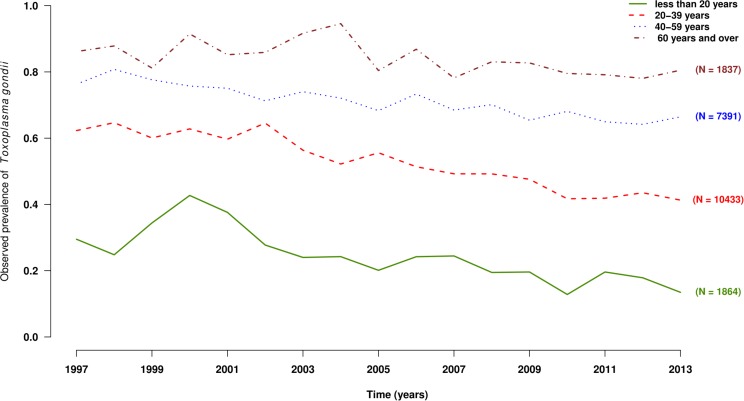
Observed prevalence of the presence of anti-*Toxoplasma gondii* antibodies by year and age groups, under 20 years old (solid curve), 20–39 years old (dashed curve), 40–59 years old (dotted curve), and 60 years old and over (dash-dotted curve), Saint-Louis Hospital, France, 1998–2013.

**FIGURE 3:**
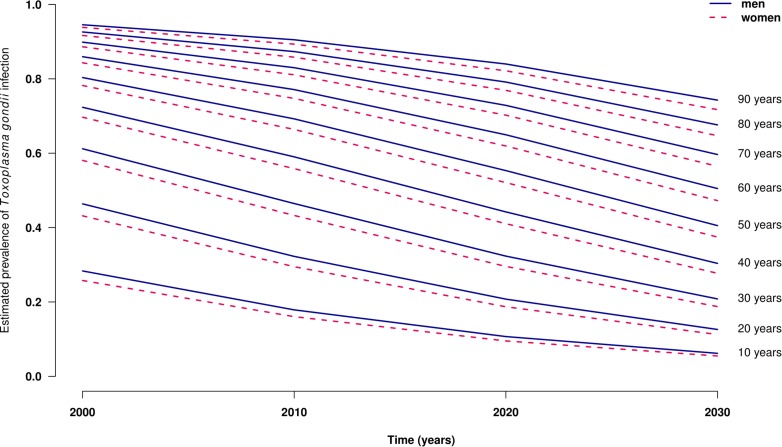
Estimated seroprevalence of *Toxoplasma* infection by age and year among men (solid curve) and women (dashed curve), Saint-Louis Hospital, France, 2000–2030.

A total of 21,480 results were analyzed [i.e., 1264 +/- 116 tests per year without significant difference according to year (**Table 1**)]. These results corresponded to 11,994 men and 9,486 women (M/F ratio: 1.3). Globally, 8,567 (39.9%) results were negative, 12,622 (58.8%) were positive, and 291 (0.7%) were equivocal using the Platelia toxo IgG. These 291 results were all positive or equivocal using the confirmatory test, leading to a global positive result number of 12,913 (60.1%). The seroprevalence increased with the age of the patients, from 20% under 20 years to more than 80% after 70 years (**Figure 1**). On the other hand, the seroprevalence continuously decreased over time between 1997 and 2013 (**Figure 2**) from 64.5% in 1997 to 54.7% in 2013 (i.e., -1.3% per year, *p* < 0.001). This drop was much more pronounced for patients aged <20 years, with an annual drop of 5.0% compared to patients aged >60 years (0.5% per year). For each gender and age, time-dependent prevalence was estimated using a logistic regression model. For both sexes, the model predicts that the prevalence will continuously decrease for all ages with higher rates in younger ages (**Figure 3**).

**Table 1:** Prevalence of anti-*Toxoplasma gondii* antibodies (%) by year and sex.

Year	1997	1998	1999	2000	2001	2002	2003	2004	2005	2006	2007	2008	2009	2010	2011	2012	2013
Male patients (*N*)	993	724	743	625	657	720	625	685	704	708	662	709	681	670	704	710	674
Prevalence (%)	67.0	69.6	68.9	71.4	66.4	64.4	63.7	62.5	59.2	62.4	59.7	59.0	59.8	61.2	58.2	58.2	54.2
Female patients (*N*)	639	636	599	561	605	618	533	534	569	575	484	483	513	535	526	583	493
Prevalence (%)	60.6	63.5	60.1	62.7	61.5	62.8	54.4	54.5	54.3	56.3	53.7	55.5	51.7	51.4	51.5	50.4	55.4
All patients (*N*)	1632	1360	1342	1186	1262	1338	1158	1219	1273	1283	1146	1192	1194	1205	1230	1293	1167
Prevalence (%)	64.5	66.8	65.0	67.3	64.0	63.7	59.4	59.0	57.0	59.7	57.2	57.6	56.3	56.8	55.4	54.7	54.7

In the multivariate analysis (**Table 2**), we found a significant association with sex (*p* < 0.001). When grouping the patients by age, there was a significant difference between the proportion of seropositive men and women in the 15- to 45-year-old group (58.5% versus 52.0%, *p* < 10^-3^), a difference which was no longer significant if ages were <15 (*p* = 0.99) or >45 years (*p* = 0.48).

**Table 2:** Logistic regression model performed to estimate *Toxoplasma gondii* prevalence, France, Saint-Louis hospital, 1997–2013.

(A)				
**Variable**	**Fractional polynomial Transformation** η(*a*)	**Regression coefficient estimate (standard error)**	***p*-Value**	**95% Confidence interval**
Age	(*age*/10^-0.5^ - 1.95	1.88 (0.04)	<0.001	1.81–1.97
Time	time - 2004.75	-0.06 (0.00)	<0.001	-0.07–0.05
Sex		0.13 (0.03)	<0.001	0.07–0.19
Intercept		0.46 (0.02)	<0.001	0.41–0.50

$\begin{array}{l} Model\;\exp ression:\\ logit\;(P(Y=1|a\text{, }t\text{, }s)=1.88\left[{\left(\frac{a}{10}\right)}^{-0.5}-1.95\right]-0.06[t-2004.75]-0.13s+0.46 \end{array}$, for age *a* and sex *s* at time *t*.				

**(B)**				
**Variable**	**Odds ratio** **(OR)**		**95% Confidence interval**	

**Age**				
5	0.09		(0.08–0.10)	
10	0.15		(0.14–0.16)	
15	0.23		(0.22–0.25)	
20	0.33		(0.31–0.35)	
25	0.45		(0.44–0.47)	
30	0.60		(0.59–0.62)	
Ref: 40	1.00			
60	2.34		(2.25–2.43)	
80	4.79		(4.47–5.13)	
**Time**				
Ref: 1997	1			
2000	0.84		(0.82–0.85)	
2003	0.70		(0.68–0.73)	
2005	0.63		(0.60–0.65)	
2010	0.47		(0.43–0.50)	
2013	0.39		(0.36–0.43)	
Sex	1.14		(1.07–1.21)	
Intercept	1.58		(1.51–1.65)	

The prevalence in women of childbearing age (15–45 years) was individualized in 2010 and 2013 to allow comparison with data provided by studies on pregnant women in France (Nogareda et al., 2014; Tourdjman et al., 2015). The prevalence was 53.5% (212/396) in 2003 and 39.7% (136/343) in 2010 which were in the same order than the figures published in 2003 (47–52.75%) and in 2010 (41.25–47%) in the 15- to 45-year-old women (Tourdjman et al., 2015). The seroprevalence projections in 2020 for the 20- to 40-year-old women with the present data are provided in **Table 3**. When compared with projections previously published with data of French pregnant women (Nogareda et al., 2014), the estimated seroprevalence was superior of 2.4% in the present study (**Table 3**).

**Table 3:** Estimated seroprevalence of anti-*Toxoplasma gondii* antibodies in 2020 from our study and from Nogareda et al. (2014) [7].

Age (year)	Present study (%)	French pregnant women (%)	Differences (%)
20	18.7	17.7	1.0
25	24.0	22.2	1.8
30	29.6	26.9	2.7
35	35.3	32.1	3.2
40	41.0	37.8	3.2

## Discussion

The present study comprising 21,480 analyses over 17 consecutive years showed a progressive and constant decrease over time of the prevalence of anti-*Toxoplasma* IgG, from 64.5% in 1997 to 54.7% in 2013 (i.e., a mea decline of 1.3% per year) with an accelerating decrease for patients aged <20 years. The projection predicts a continuous decrease until 2030 for all age groups (**Figure 3**).

In France, a country with one of the highest rates of toxoplasmosis (Pappas et al., 2009), sectional studies relying on screening of pregnant women show that *Toxoplasma* infection prevalence has markedly decreased from 83% in 1965 to 54% in 1995 and 44% in 2003 (Berger et al., 2009), and 37% in 2010 (Nogareda et al., 2014). When considering the data from pregnant women of the Paris area, one of the French regions with the highest seroprevalence of toxoplasmosis, the figures published in 2003 (47–52.75%) and in 2010 (41.25–47%) are very similar to the seroprevalence values observed in 2003 (53.5%) and in 2010 (39.7%) in the 15- to 45-year-old women of the present study (Tourdjman et al., 2015). Similarly, the estimated prevalence in 2020 in our study (**Table 2**; **Figure 3**) is similar to the one previously proposed using data on pregnant women (Nogareda et al., 2014) taking into account the higher prevalence in the Paris area compared with other parts of France. Both models predict a constant decline in seroprevalence. Thus, our present data confirm the results obtained using cross-sectional seroprevalence studies of pregnant women (Berger et al., 2009; Nogareda et al., 2014).

The similar range of seroprevalence between patients and women of child-bearing age of our study is in disagreement with the recent analysis showing a higher prevalence in immunocompromised patients compared to controls (Wang et al., 2017). The estimated pooled prevalence of *T. gondii* infection in cancer patients was 26.0 and 12.1% for the control group (*p* < 0.001), and 42.1 in transplant recipients versus 34.5% for the control (*p* < 0.05) (Wang et al., 2017). This publication shows huge differences in the control groups, who should represent the general population, suggesting that pooling data from different countries with different baseline seroprevalence should be done with caution. In a given setting, the seropositivity represents the risk to have reactivation, and by definition is associated with toxoplasmosis, as underlined by Wang et al. (2017), since reactivation is the main mechanism of occurrence of toxoplasmosis (Martino et al., 2000). But serology used for diagnosis of infection during immunosuppressive therapy is hampered by weak and altered immune response and blood transfusion, which rises the risk of detecting donor antibodies. Nowadays, diagnosis is more reliably assumed by detecting the parasite using PCR (Bretagne et al., 2000; Martino et al., 2005). However, we cannot exclude that, once the patient is immunocompromised because of treatment or HIV infection, they become more at risk of contracting toxoplasmosis after eating contaminated food to explain the higher prevalence observed in cancer patients and transplant recipients compared with the general population (Wang et al., 2017).

The seroprevalence is known to vary by country, from <10% in China to >60% in Brazil (Pappas et al., 2009). Indeed, *T. gondii* infection is subject to complex environmental, socioeconomic, and dietary habits and can change over time. However, a decline in seroprevalence has been reported from several databases in many countries regardless of the initial seroprevalence. A decline among United States-born persons aged 12–49 years from 14.1 to 9.0% comparing data from 1988 to 1994 and 1999 to 2004 (i.e., a 38.8% reduction) was reported based on the National Health and Nutrition Examination Surveys (Jones et al., 2007). A population-based study performed in northern Greece reported a continuous decline, from 37 to 29.9 and 24.1% in 1984, 1994, and 2004, respectively (Diza et al., 2005). In Argentina, the seroprevalence in blood donors declined from 67% in 1967 to 21.2% in 2017 (i.e., an average annual decline of 0.9%) (Kaufer et al., 2017). In Austria, the seroprevalence in women of childbearing age decreased from 43.3% in 1995 to 31.5% in 2012, with a decline of 0.84% per year (Berghold et al., 2016). A 6-year study of pregnant women in Poland showed a decreased incidence, from 45.4% in 1998 to 39.4% in 2003, with an annual decline of 1.0% (Nowakowska et al., 2006).

Keeping the route of transmission of *T. gondii* in mind (Dubey and Jones, 2008), this decline could be explained by a decrease in the ingestion of salads and fresh vegetables contaminated by oocysts excreted by cats. A « Fédération des fabricants d’aliments préparés pour chiens, chats, oiseaux et autres animaux familiers » (FACCO) 2016 study including 14,000 French households showed a significant increase in the cat population in France, from 10 to 13.5 million between 2006 and 2016^3^. Since the seroprevalence decreases in humans, if cats were the main source of human contamination, this would imply that fewer cats are being contaminated (Cook et al., 2000). Indeed, domestic cats are largely fed with dry food and are therefore less susceptible to ingesting raw meat from the hunting of rodents in contrast to rural cats living in areas with high farm densities, which have a seroprevalence >70% (Afonso et al., 2013). One might also argue that the recommendation to wash crude vegetables as part of the prevention program for pregnant women could account for a portion of the decline in seroprevalence. Drinking water contaminated with oocysts is another possible source of infection but no French data on oocyst contamination of drinkable water is currently available. An increase in the consumption of bottled water could have also reduced the occurrence of oocyst ingestion along with improvement of quality of tape water.

However, the foremost explanation of the general decline of seroprevalence is likely the decrease of consumption of infected meat over time. Indeed, the progressive increase of seroprevalence according to patient age is highly suggestive of the cumulative risk secondary to ingestion of infected meat, the probable main route of infection in Europe (Cook et al., 2000). Therefore, the decline in seroprevalence over time can be explained by both the decrease of consumption of meat and the decrease of parasites in commercialized meat. The consumption of mutton, the main meat containing *T. gondii* cysts, has declined from 5.4 to 2.7 kg/inhabitant/year between 1990 and 2013 in France, either a 50% drop. Additionally, mutton is more and more imported from countries such as the United Kingdom where the prevalence of *Toxoplasma* is lower than in France^4^. Deep-freezing during transportation that kills tissue cysts may also contribute to decreased human exposure to *Toxoplasma*. On the other hand, the organic method of reared meat production, with animals free to roam outdoors, could reverse this trend by increasing the risk of meat contamination from environmental sources (Guo et al., 2015).

A particular observation of the present study is the higher rate of seroprevalence in men versus women between 15 and 45 years of age. This higher seroprevalence in men has already been noted in France (45.3 vs. 41.6%) (Bellali et al., 2013) and in the United States (11.6 vs. 9.9%) (Jones et al., 2007) although the sample size was too small to be conclusive. In Argentina, the difference between men and women was not statistically significant in data from 1997 and 2007 but significant (*p* < 0.001) in 2017 based on a study of 1,393 blood donors (Kaufer et al., 2017). A possible explanation could be the prevention program geared toward pregnant women to decrease the ingestion of undercooked meat–information widely provided to women of childbearing age in France. However, this hypothesis should be investigated further and other factors could explain the observed sex difference, such as higher meat consumption in men compared to women or a bias in the population presently studied.

Our study had several limitations that should be taken into account. The decrease in seroprevalence could have been related to variations in the patient populations. The general recruitment of our hospital has not been dramatically modified for the last 10–15 years but the underlying disease could not be obtained from the laboratory and we cannot exclude modifications of the recruitment as a cause of decreased prevalence. Another limitation is that the geographical origin of the patients was not available, nor were their culinary habits, which would have provided added context for interpreting the decrease of seroprevalence. However, the comparison with previous data independently collected from pregnant women showed similar trends. Moreover, an advantage of our data is to have been provided by using the same screening commercial kit and thereby limiting variation in multiple kit use that is commonly found in the literature (Chemoh et al., 2013). Other biases could be discussed for the estimation of the seroprevalence values. For instance, although the serodiagnosis was performed before any chemotherapy or graft procedure, many patients could have received blood derivatives before hospitalization, which could have impacted the serology results (Foroutan-Rad et al., 2016). We also considered as positive patients with equivocal results considering that the best message for the clinicians was to increase the suspicion index for the risk of reactivation. This overestimates the positivity rate of 0.7% compared with pregnant women for who the equivocal results are considered negative to continue the surveillance during pregnancy. Thus, our data confirm previous observations in women of childbearing age although the present values are in the highest range of the French seroprevalence values.

Our projection for the near future shows that for immunocompromised patients, the risk of reactivating *Toxoplasma* cysts will likely become increasingly rarer–although still possible–in pediatric patients (Decembrino et al., 2017), with a prevalence below 20% predicted in 2020 for those under 20 years of age. On the other hand, older patients are predicted to remain at high risk of reactivation, with a seroprevalence expected to be >60% in 2020 and >47% in 2030 in those aged 60 years, and >52% in 2020 and >37% in 2030 in those aged 50 years (**Figure 3**). Therefore, we advocate for continuous screening of immunocompromised patients before starting any specific therapy. This screening allows focusing PCR investigation in patients at risk of reactivation when no prophylaxis is present or to investigate fevers of unknown origin in immunocompromised patients (Costa et al., 2000).

The seroprevalence of toxoplasmosis measured in the present study shows a downward trend similar to the one observed by the national perinatal surveys regularly performed in France and in other countries. The reasons for this continuous decline are likely linked to the lower parasitism of consumed meat (mainly mutton) due to improved sanitation in the preparation and transportation of animal carcasses as well as more sterile culinary habits in general. To continue performing serology tests in immunocompromised patients seems to us important in order to categorize patients according to the risk of cyst-induced reactivation, especially in older patients.

## Author Contributions

SB and AA conceived and designed the study. NG, SH, and MG-M performed data collection. NG, LL, and YLS analyzed the data. SB, LL, and NG wrote the paper. All authors read, commented, and approved the final manuscript.

## Conflict of Interest Statement

The authors declare that the research was conducted in the absence of any commercial or financial relationships that could be construed as a potential conflict of interest.

## Footnotes

1 https://www.has-sante.fr/portail/upload/docs/application/pdf/2017-07/dir152/inahta_brief__toxoplasmosis.pdf

2 https://www.has-sante.fr/portail/upload/docs/application/pdf/2015-12/rbp_recommandations_greffe_renale_vd_mel.pdf

3 https://www.facco.fr/les-chiffres/

4 http://www.interbev.fr/wp-content/uploads/2015/10/LIVRET-VIANDES-OVINE-2015-BD.pdf
