# Corrigendum: Continuous Decline of *Toxoplasma gondii* Seroprevalence in Hospital: A 1997–2014 Longitudinal Study in Paris, France

**DOI:** 10.3389/fmicb.2018.02814

**Published:** 2018-11-27

**Authors:** Nicolas Guigue, Lucie Léon, Samia Hamane, Maud Gits-Muselli, Yann Le Strat, Alexandre Alanio, Stéphane Bretagne

**Affiliations:** ^1^Laboratoire de Parasitologie-Mycologie, AP-HP, Groupe Hospitalier Saint-Louis, Lariboisière, Fernand-Widal, Paris, France; ^2^Santé Publique France, French National Public Health Agency, Saint-Maurice, France; ^3^Université Paris Diderot, Sorbonne Paris Cité, Paris, France

**Keywords:** *Toxoplasma gondii*, serology, prevalence, immunocompromised patient, France

In the original article, the order of the affiliations was incorrect. The correct order appears above. In addition, the city for affiliation 2 should be Saint-Maurice instead of Paris. The authors apologize for this error and state that this does not change the scientific conclusions of the article in any way. The original article has been updated.

## Conflict of interest statement

The authors declare that the research was conducted in the absence of any commercial or financial relationships that could be construed as a potential conflict of interest.

